# Intervention of Dietary Protein Levels on Muscle Quality, Antioxidation, and Autophagy in the Muscles of Triploid Crucian Carp (*Carassius carassius Triploid*)

**DOI:** 10.3390/ijms241512043

**Published:** 2023-07-27

**Authors:** Zhimin He, Yuyang Cai, Yang Xiao, Shenping Cao, Gaode Zhong, Xinting Li, Yanfang Li, Junhan Luo, Jianzhou Tang, Fufa Qu, Zhen Liu, Suchun Liu

**Affiliations:** 1 Hunan Provincial Key Laboratory of Nutrition and Quality Control of Aquatic Animals, College of Biological and Chemical Engineering, Changsha University, Changsha 410022, China; z20180831@ccsu.edu.cn (Z.H.); caiyuyang@stu.hunau.edu.cn (Y.C.); z20151114@ccsu.edu.cn (F.Q.); 2 College of Food Science and Technology, Hunan Agricultural University, Changsha 410128, China

**Keywords:** *Carassius carassius triploid*, dietary protein level, muscle quality, antioxidation, autophagy

## Abstract

The aim of this study is to investigate the effect of dietary protein levels on flesh quality, oxidative stress, and autophagy status in the muscles of triploid crucian carp (*Carassius carassius triploid*), and the related molecular mechanisms. Six experimental diets with different protein levels (26%, 29%, 32%, 35%, 38%, 41%) were formulated. A total of 540 fish with an initial weight of 11.79 ± 0.09 g were randomly assigned to 18 cages and six treatments with three replicates of 30 fish each for 8 weeks feeding. It could be found that the whole-body ash content significantly increased in high protein level groups (*p* < 0.05). The 29% dietary protein level group exhibited the highest muscle moisture, although there was an inconspicuous decrease in the chewiness of the muscles when compared with the other groups. The dietary protein level influenced the content of free amino acids and nucleotides, especially the content of flavor amino acids, which exhibited an increasing tendency along with the increasing protein level, such as alanine and glutamic acid, while the flavor nucleotides showed different fluctuation trends. Moreover, the genes related to muscle development were shown to be influenced by the dietary protein level, especially the expression of *MRF4*, which was up-regulated with the increasing dietary protein levels. The 29% dietary protein level promoted the majority of analyzed muscle genes expression to the highest level when compared to other dietary levels, except the *Myostain*, whose expression reached its highest at 38% dietary protein levels. Furthermore, the effect of dietary protein levels on antioxidant signaling pathway genes were also examined. High protein levels would boost the expression of *GSTα*; *GPX1* and *GPX4α* mRNA expression showed the highest level at the 32% dietary protein group. The increasing dietary protein level decreased both mRNA and protein expressions of Nrf2 by up-regulating *Keap1*. Autophagy-related gene expression levels reached the peak at 32% dietary protein level, as evidenced by a similar change in protein expression of FoxO1. In summary, muscle nutritional composition, antioxidative pathways, and autophagy levels were affected by the dietary protein levels. A total of 29–32% dietary protein level would be the appropriate level range to improve muscle quality and promote the antioxidant and autophagy capacity of triploid crucian carp muscles.

## 1. Introduction

Protein is a crucial component in the diets of aquatic animals; its source, inclusion level in feed, and quality can significantly impact various aspects of reared fish. The effects of the protein source on fish growth performance, flesh quality, and antioxidant capacity have received considerable attention. However, the inclusion level of protein in feed is an effective means of controlling production costs, and it has also been shown to influence muscle quality in several aquatic animals. For example, the protein inclusion level has been found to affect the growth performance and flesh quality of the fish, as well as alter the antioxidant capacity of grass carp [1]. In grass carp, muscle hardness is influenced by the dietary protein level, which affects the growth of muscle fibers as well as the synthesis and degradation of collagen [2]. In *kuruma shrimp*, a low dietary protein level has been shown to slow down the growth rate, decrease the intestinal trypsin activity, and affect the hardness [3]. Furthermore, studies have indicated that a 45% protein dietary was more susceptible to oxidative stress than the 55% protein dietary group for Senegalese sole juveniles (*Solea senegalensis*) [4]. Both dietary protein level and origin have been found to influence the antioxidative defense status in rats [5]. A high-protein diet can lead to an imbalance between oxidants and antioxidants, resulting in oxidative stress in digestive organs of mice [6].

It has also been discovered that oxidative stress can have irreversible deleterious effects on muscle quality. When animals are stimulated by the oxidation of fish oil in feed [7], a high-carbohydrate [8], or high-fat [9] diet, as well as a high-temperature environment, the production and scavenging of reactive oxygen radicals in the body can become imbalanced, leading to oxidative damage to tissues and cells. The polyunsaturated fatty acids in muscles easily combine with reactive oxygen radicals, resulting in the production of lipid peroxides, particularly malondialdehyde (MDA); the aldehyde group of which can combine with protein residues to activate protein oxidation, resulting in amino acid dysfunction, protease inactivation, and other negative effects, thereby compromising muscle nutrition and quality. For example, the inactivation of μ-calcium protease decreases the amount of myofibrillar protein hydrolysis, resulting in reduced meat tenderness [5,10]. Additionally, excessive accumulation of reactive oxygen species can also cause DNA damage in myocytes and activate poly ADP-ribose polymerase 1, which can impair meat tenderness by catalyzing the degradation of myogenic fibronectin [11]. Therefore, reducing the oxidative stress in the body and enhancing the defensive capacity of the antioxidant system are critical for improving muscle quality.

Autophagy is the ability of intracellular lysosomes to degrade their own components, which is a highly conserved process. It is activated under conditions of nutrient limitation [11], copper exposure [12], pathogenic infections [13,14], and other stress responses, leading to the formation of autophagic vesicles for the removal of functionally impaired intracellular membranes, organelles, and other materials. Thus, the level of autophagy can be used as an indicator, which can reflect the health status of the organism. It has been found that reactive oxygen species (ROS) can activate mitochondrial autophagy to remove damaged mitochondria after the body is exposed to oxidative stress. However, excessive ROS could damage mitochondrial function, forming a vicious cycle [15]. There appears to be a relationship between muscle quality, oxidative stress, and autophagy signaling molecule expression. The addition of antioxidants to the diet can significantly increase the content of polyunsaturated fatty acids in muscle healthcare substances, upregulate the expression of genes related to muscle fiber development to improve meat hardness, upregulate *Nrf2* to activate antioxidant enzymes, and inhibit LC3 protein expression to alleviate oxidative stress and autophagy [1].

Protein is the most essential component in animal diets, and variations in dietary protein levels can dramatically affect growth performance through the GH-IGFs axis and TOR/S6K1 pathway [16]. Yet, we found in a significant number of studies on dietary protein levels that dietary protein levels also have a crucial role in controlling animal muscle quality and antioxidant status. Animals need to accelerate the formation of ATP to replenish energy due to enhanced protein metabolism, and the amount of ROS, a by-product of ATP formation, increases significantly, inducing oxidative damage. Recent findings in grass carp reveal that diets with different protein levels regulate antioxidant capacity and improve muscle texture and flavor, primarily by regulating the muscle Nrf2-Keap1 signaling pathway, which mediates antioxidant enzyme activity and gene expression [3]. Autophagy is inextricably linked to oxidation, and since autophagy is an organism’s response behavior to mitigate oxidative stress, its level might also change depending on the degree of oxidative damage in the organism. As a consequence, investigating the regulation of muscle development, antioxidant, and autophagy by different protein levels at the molecular level is a useful method for determining the optimal protein level of feed, which is a good way to optimize feed formulation for animals. However, there is little information on the interacting effects of dietary protein level on muscle quality, antioxidation, and autophagy to our knowledge.

Triploid crucian carp is a kind of polyploidy fish (3n) with several advantages, such as high growth rate, greater stress tolerance, and enhanced immune response [8,9,11,12,15,17]. Triploid crucian carp (Xiangyunji) used in this study were produced by mating male *allotetraploids* with female *Carassius auratas* (*C. auratas*) [10]. Compared with the parents, it has some change in superiority in biological characteristics, such as the higher body, shorter tail, smaller head, as well as a higher meat yield [18]. Furthermore, under stress conditions, triploid crucian carp showed greater stress resistance than diploid crucian carp [17]. It is necessary to improve the muscle quality through feed formulation improvement in order to further increase the economic value of triploid crucian carp.

Although the protein requirements have been studied extensively, little information about the protein requirement of triploid crucian carp is available. The present work is being conducted to evaluate the dietary protein inclusion levels for triploid crucian carp by studying flesh quality, including nutritional composition, amino acid composition, and texture properties of the muscles and exploring its regulatory mechanisms related to muscle quality, antioxidation, and autophagy, which may provide a more theoretical foundation for improving muscle quality and the immunity of triploid crucian carp by nutritional strategies.

## 2. Results

### 2.1. Effects of the Dietary Protein Levels on Muscle Quality of Triploid Crucian Carp

#### 2.1.1. Effects of the Dietary Protein Levels on Body and Muscle Composition of Triploid Crucian Carp

The effects of dietary protein levels on whole-body and dorsal muscle composition of triploid crucian carp are shown in Table 1. The whole-body ash content of fish fed with a dietary protein level 35% or more displayed higher ash content than other groups (*p* < 0.05) (Table 2). Fish in the group of 29% owned higher muscle moisture content than other groups. The dietary protein levels were investigated to have no influence on the moisture and crude lipid of whole body, as well as the ash and crude protein of triploid crucian carp muscle as presented in Table 2. In addition, the different protein level caused no significantly changes in condition factor (CF). The hepatosomatic index tended to decrease with the protein inclusion levels from 26 to 35% and then increased slightly at 38%, finally sharply decreased to the bottom at 41%, but there was no significant difference among the different dietaries (*p* > 0.05) (Table 2).

#### 2.1.2. Effects of Dietary Protein Levels on the Amino Acid and Flavor Nucleotide 

##### Content of Muscle

As shown in Table 3, flavor amino acid content fluctuated with the increasing of dietary protein level, but generally tended to increase and then decrease, peaking at the 29% level. While the glutamic in the muscles of fish fed with a high-protein diet was overall higher than that in fish fed with a low-protein diet. No significant differences of muscle alanine, tyrosine, or asparagine content was observed in different groups. Dietary protein levels effect on the other free amino acid of muscle analysis showed that histidine content was positively regulated by the dietary protein level and owned a liner relationship with dietary protein level. Other free amino acids presented in Table 3 except the methionine were observed to have significant differences among different groups and their content would reach a peak at an appropriate dietary protein level. 

**Figure 1:**
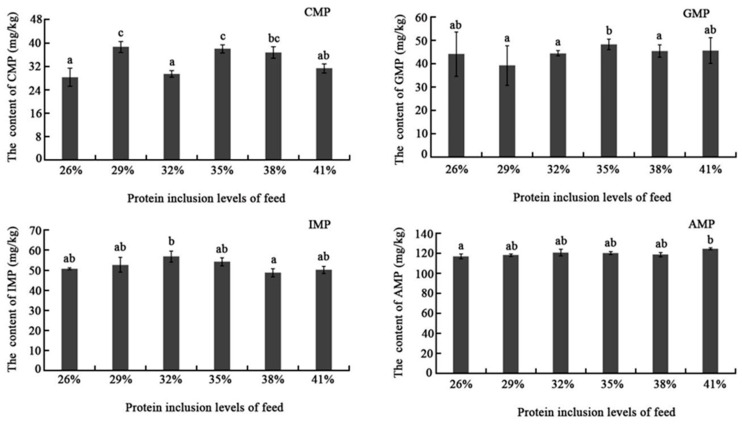
Effects of different dietary protein inclusion levels on flavor nucleotide content in muscles of triploid crucian carp. Data are represented as the mean values ± SEM (n = 3 repetitions for each group with a total 3 × 3 fish) and were analyzed by *t*-test. The different lowercase letters represent a significant difference between the two comparison groups (*p* < 0.05).

The content of flavor nucleotide in muscle including CMP, GMP, IMP, and AMP were slightly affected by the dietary protein level (Figure 1). CMP showed a relatively obvious increasing tendency.

#### 2.1.3. Effects of Dietary Protein Levels on the Texture of Muscle

**Figure 2:**
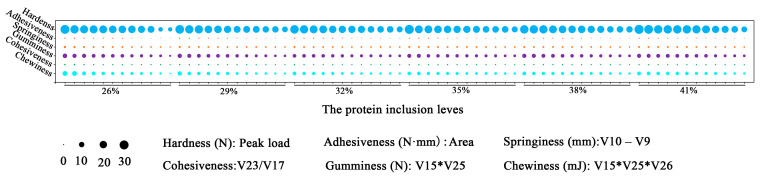
Bubble chart of muscle texture properties. Notes: Results presented in the chart are expressed as means ± S.E. (n = 6) and were analyzed by *t*-test. Hardness was presented in the form of peak load (V15). Adhesiveness was presented in the form of peak area between the two compressions. Springiness was presented in the form of difference between two compression length (V10–V9). Gumminess was presented in form of hardness*cohesiveness (V15*V25). Cohesiveness was presented in form of ratio of two compressed areas (V23/V17). Chewiness was presented in form of gumminess * springiness (V15*V25*V26).

Chewiness and cohesiveness tended to vary significantly (manifested as a different color) among all textural properties, and the 32% dietary protein level group decreased the chewiness and cohesiveness obviously compared to the other groups (Figure 2 and Table 4).

### 2.2. Effects of Dietary Protein Level on the Expression of Related Genes in Muscle of Triploid Crucian Carp

**Figure 3:**
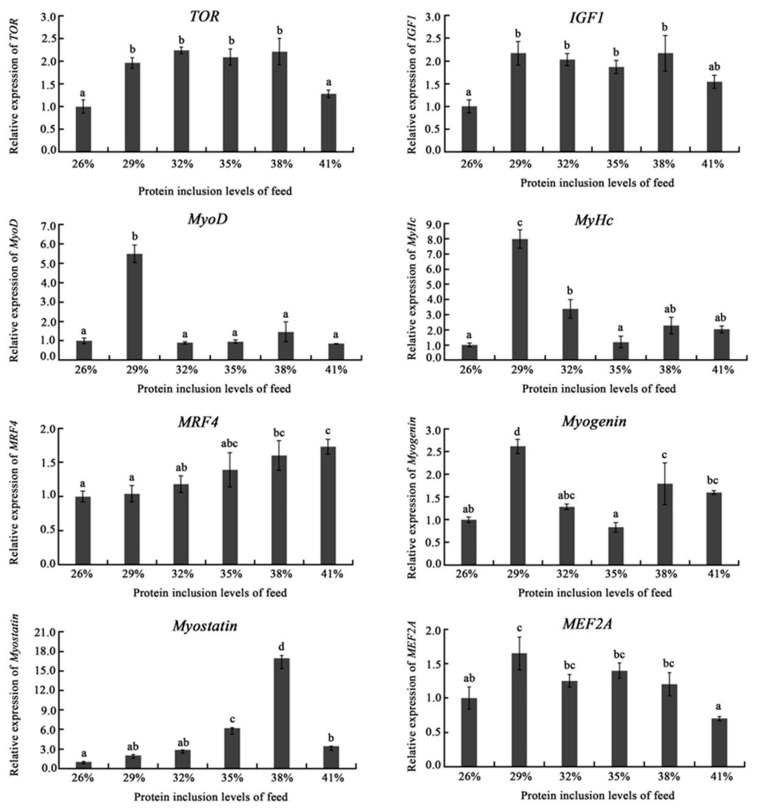
Effects of diet with different protein levels on the mRNA expression of muscle development related genes in the muscle of triploid crucian carp. Notes: Results presented in the chart are expressed as means ± S.E. (n = 3 repetitions for each group with a total 3 × 3 fish) and were analyzed by *t*-test. The different lowercase letters represent a significant difference between the two comparison groups (*p* < 0.05).

As shown in Figure 3, the dietary protein level exhibited significant influence on the expression of genes in the muscle of triploid crucian carp. The mRNA expression of *TOR*, *IGF1*, and *MEF2A* was investigated to be higher in the range of 29–38% dietary protein groups than those of them in the 26% and 41% protein dietary groups, suggesting appropriate dietary protein inclusion level range would promote their expressions. In addition, the expressions of *MyoD*, *MyHc*, and *Myogenin* showed peaking in the 29% protein dietary group, while *Myostanin* expression reached peak in the 38% dietary protein group. Moreover, the expression of *MRF4* was up-regulated with the increasing of dietary protein levels.

### 2.3. Effects of Dietary Protein Level on the Expression of Antioxidant Signaling Pathway Genes of Triploid Crucian Carp

**Figure 4:**
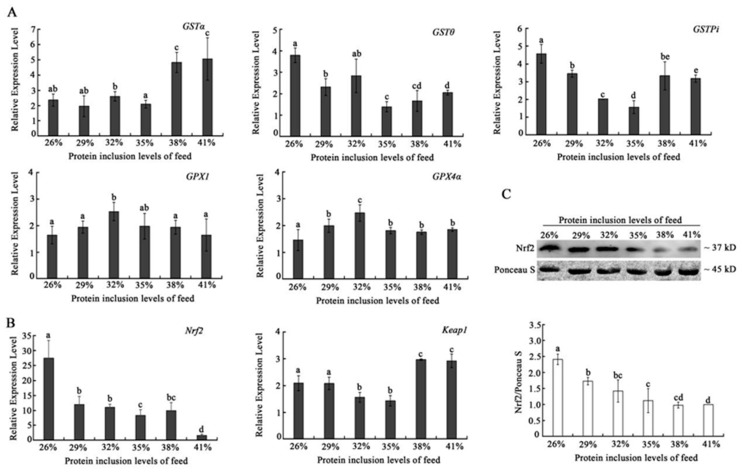
Effects of dietary with different protein levels on the mRNA expression level of antioxidant genes in the muscle of triploid crucian carp. (**A**) The mRNA expression level changes of *GSTα*, *GSTθ*, *GSTPi*, *GPX1*, and *GPX4α* with different dietary protein levels; (**B**) The mRAN expression level changes of *Nrf2* and *Keap1* with different dietary protein levels; (**C**) The protein level changes of Nrf2 protein with different dietary protein levels. Notes: Results presented in the chart are expressed as means ± S.E. (n = 3 repetitions for each group with a total 3 × 3 fish) and were analyzed by *t*-test. The different lowercase letters represent a significant difference between the two comparison groups (*p* < 0.05).

*GSTθ* and *GSTPi* showed higher expression levels in the 26% dietary protein group than others. However, 38% and 41% high dietary protein levels exhibited the promotion on the expression of *GSTα*. *GPX1* and *GPX4α* was investigated to own obviously highest expressions at 29% dietary protein group. Both the mRNA and protein levels of Nrf2 showed similar overall tendency, which descended with the increasing of dietary protein levels. *Keap1* showed an antagonistic expression tendency when compared to *Nrf2* in the high dietary protein level groups (38% and 41%) (Figure 4).

### 2.4. Effects of Dietary Protein Level on the Expression of Autophagy Status-Related Genes of Triploid Crucian Carp

**Figure 5:**
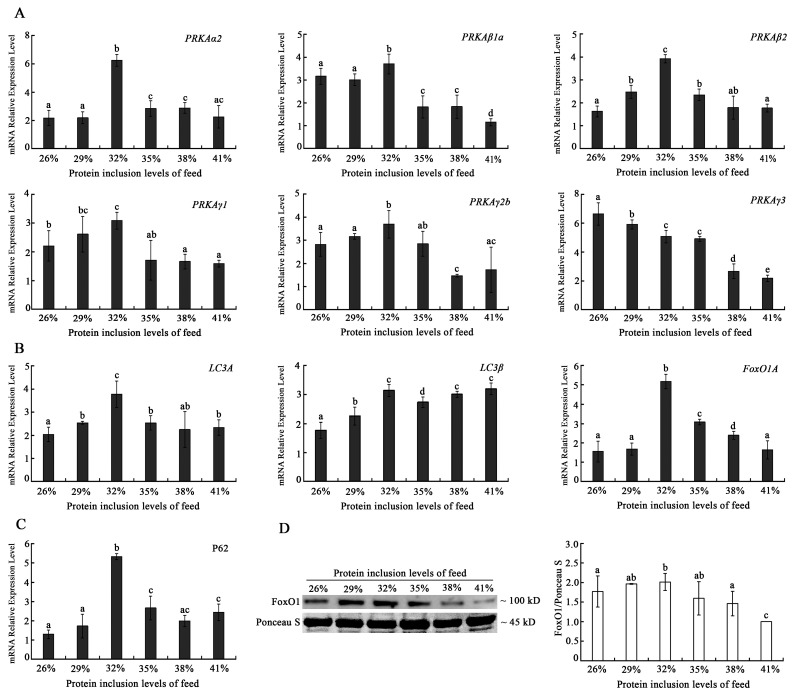
Effects of dietary with different protein levels on the autophagy in the muscle of triploid crucian carp. (**A**) The mRNA expression level changes of *PRKAa2*, *PRKAβ1a*, *PRKAβ2*, *PRKAγ1*, *PRKAγ2b*, and *PRKAγ3* with different dietary protein levels; (**B**) The mRAN expression level changes of *LC3A*, *LC3β*, and *Foxo1A* with different dietary protein levels; (**C**) The mRAN expression level changes of P62 with different dietary protein levels; (**D**) The protein level changes of FoxO1 protein with different dietary protein levels. Notes: Results presented in the chart are expressed as means ± S.E. (n = 3 repetitions for each group with a total 3 × 3 fish) and were analyzed by *t*-test. The different lowercase letters represent a significant difference between the two comparison groups (*p* < 0.05).

To examine the effects of dietary protein level to autophagy capacity of triploid crucian carp muscles, the expressions of autophagy-related genes were analyzed. The results showed that PRKA sbutypes, including PRKAα2, PRKAβ1a, PRKAβ 2, PRKAγ1, PRKAγ2b, and LC3A, LC3B, FoxO1A, and P62 mRNA expression levels as well as the protein level of FoxO1 exhibited the highest mRNA expression in the 32% dietary protein level group. Additionally, the PRKA subtype -PRKAγ3 mRNA expression was down-regulated with the increase in dietary protein levels (Figure 5).

### 2.5. Pearson’s Correlation between Dietary Protein Level and Response Variables

As shown in Table 5, the analysis of Pearson’s correlation showed that the levels of *MRF4*, histidine, alanine, isoleucine, whole body ash, *LC3β*, leucine, and *GSTα* were extremely significantly or significantly positively related to dietary protein levels. While the levels of glycine, adhesiveness, Nrf2 protein, *PRKAγ3*, HIS, *Nrf2*, *PRKAβ1α*, and FoxO1 protein were extremely significantly or significantly negatively related to dietary protein levels. Other analyzed response variables did not exhibit an obvious relationship along with the dietary protein levels.

## 3. Discussion

From a macroscopic standpoint, nutrient content could reflect fish quality [19]. Since Ca and P account for approximately 80–90% of the total minerals in fish, ash is primarily composed of inorganic elements such as potassium (K), phosphorus (P), sodium (Na), and calcium (Ca) [20]. In the current study, the ash content of triploid crucian carp carcass increased with the increasing dietary protein levels with a significant positive correlation (r = 0.896, *p* < 0.05). This increase in ash content is believed to be due to the deposition of calcium and phosphorus, although the specific regulation mechanism requires additional investigation. A recent study has demonstrated that the increasing dietary protein levels increased the water content of both the carcass and muscles while decreasing the fat content [1]. Although there was a declining trend in the crude fat content of fish carcasses in the current investigation, dietary protein level had no significant effect on the moisture and crude fat of triploid crucian carp carcasses, which is consistent with those of *Tilapia aurea* [21]. 

Moreover, protein content is a crucial factor influencing meat quality. An optimal dietary protein level could considerably improve muscle protein content [22]. In this study, the muscle’s crude protein content of triploid crucian carp had no obvious significance between groups cultured with different protein inclusion levels feed, but the moisture content significantly increased in the group with a 29% dietary protein level, suggesting that dietary protein level might affect muscle quality by increasing the moisture content, but the specific taste quality needs to be further investigated in conjunction with the texture characteristics. On the other hand, a dietary protein level of 28–58% was investigated to have no effect on nutritional content such as crude protein, crude fat, and moisture content of the carcass and muscles [23]. Thus, different effects on nutrient content caused by varied dietary protein levels could be explained by the difference of fish species, feed, and environment [24]. Furthermore, the variation of muscle flavor substance content in triploid crucian carp was examined to investigate the influence of different dietary protein levels on muscle quality.

It is widely known that free amino acids in muscle not only serve as raw materials for muscle protein synthesis, but also act as a flavor precursor substances that have an important impact on the flavor presentation of muscles. Six amino acids—glycine, glutamic acid, aspartic acid, tyrosine, phenylalanine, and alanine—are usually thought to contribute specifically to food deliciousness. It has been shown that optimal dietary protein could improve the flavor of muscle by changing the content of free amino acids [2,25]. The overall flesh flavor amino acids were increased with dietary protein inclusion levels 38–41% in triploid crucian carp, which was consistent with a previous study in which the content of free glutamate and glycine in muscles increased linearly with dietary protein inclusion levels [26]. However, the glycine acid content declined with protein inclusion levels in triploid crucian carp. These results reveal that increasing the protein inclusion level generally upregulated the amount of free amino acids of muscles to enhance muscular flavor, but not each kind of them. 

Nucleotides could amplify sweetness and meatiness, and block or remove bitterness, sourness, and other unfavorable flavors. Nucleotides could also have a synergistic impact with free amino acids to dramatically increase flesh flavor. Until now, the impact of dietary protein inclusion level on muscle nucleotide content has not yet been extensively studied. In this study, flavor nucleotides showed no significant difference among different dietary protein inclusion levels except the CMP, which showed higher content in the 29% and 35% groups than others. This could be the consequence of rapid nucleotide degradation, but exact reason needs to be further explored. 

Textural characteristics provide a reliable foundation for assessing the quality of a muscle by simulating the chewing process of food in the mouth, including springiness, hardness, chewiness, and so on. According to widespread consensus, customers prefer fish with a crispier texture and one that is firmer, more elastic, more chewable [27]. The results of this study demonstrated that the dietary protein inclusion levels affected the chewiness and cohesiveness. Both chewiness and cohesiveness showed similar change tendencies which first declined to the bottom in the 32% group and then slightly increased. The flesh texture might exhibit delicate and non-loose qualities, but more investigation is needed in conjunction with information on water distribution and drip loss. Moreover, it is necessary to investigate the causes of the change in muscle texture features in the 32% protein level group.

The development of muscle fibers largely determines muscle quality. The myogenic regulatory factor (MRF) family genes including *MyoD*, *Myogenin*, *MRF4*, and *Myf5* are responsible for regulating the transcription of key myogenic genes [28]. *Myostanin* inhibits muscle growth, whereas *IGF1* and *MEF2A* promote it [29]. Dietary protein levels might activate *TOR* signaling pathways to stimulate muscle growth [30]. In this study, the mRNA expressions of protein synthesis-related gene-*TOR*, *IGF1*, and *MEF2A* showed high expression in the range of 29–38% dietary protein inclusion levels; *MyoD*, *MyHc*, and *Myogenin* genes exhibited the highest expression in the 29% group. In addition, *MRF4* expression improved with the increasing of dietary protein levels; while *Myostanin* expression was demonstrated to be highest in the 38% group, which may cue that 38% dietary protein inclusion level would inhibit the growth of muscle. Thus, appropriate dietary protein levels would stimulate the expression of genes related too muscle growth and development.

The organism always maintains its health by strengthening the antioxidant system’s defense to deal with ROS produced by protein metabolism and prevents oxidative damage to muscle cells. Previous studies reported that the antioxidant system was enhanced mainly by upregulating the mRNA expressions of genes, such as *GSH*, *GST*, and *GPX*, increasing the activity of enzymes, such as SOD and GSH, for scavenging oxidative metabolites ROS and MDA [25,31]. In this study, *GPX* genes (*GPX1* and *GPX4α*) expression showed a similar tendency to reach the peak in the 32% dietary protein inclusion group. While GST genes exhibited different expression tendencies with the increasing of dietary protein level, the *Nrf2*-keap 1-ARE signaling pathway is the most important endogenous antioxidant signaling pathway in current organisms [32]. *Nrf2* is the most important transcription factor in the cell oxidative stress pathway and can increase the expression level of a variety of antioxidant proteins and phase detoxification enzymes by interacting with antioxidant response elements, thereby removing free radicals produced by oxidative stress in the body so as to maintain the redox state of cells [33,34]. Both mRNA and proteins of triploid crucian carp *Nrf2* declined with the increase in dietary protein inclusion level, which suggests that the increasing dietary protein inclusion level would reduce the antioxidant level of triploid crucian carp. This result was further verified by the expression of keap1, which improved with the increasing of dietary protein inclusion level. These results indicate a negative relationship between *Nrf2* and *Keap1*, and suggest that a high dietary protein level would affect the pathway of *Nrf2*-Keap1 pathway, leading to a decline in the antioxidant capacity of the muscles of triploid crucian carp.

While under stress, mechanisms of organism protection are triggered, and a mild rise in autophagy might help cells remove damaged organelles and speed up metabolism. However, a high level of autophagy could result in stress loss, metabolic disorder, and even death of cells. The mRNA levels of autophagy markers, such as the *PRKA* and *LC3* family genes exhibited a general tendency of initially rising and subsequently falling with the increasing dietary protein levels, with the majority of them reaching their peak expression levels in the 32% dietary protein inclusion level group. FoxO1 is not only an important autophagy-induced protein, but also involves the regulation of muscle fiber development and antioxidation [35,36,37]. Due to the association between FoxO1 and autophagy, this study analyzed the mRNA and protein expression of FoxO1, which showed a similar change tendency as those of autophagy markers. Combining the antioxidant function results, however, revealed that the autophagy levels significantly increased at the 32% protein level, and when combined with the previous hypothesis of impaired regulation of Nrf2-Keap1 antioxidant function at this level state, we determined the possibility of oxidative damage in muscle cells in the 32% protein level group, implying that our previous decrease in muscle mass levels may be related to the increase in autophagy level. Furthermore, we thought that the downregulation of autophagy-related gene expression in the 38–41% protein level group was a passive rather than an active cytoprotective downregulation. It meant the autophagy status was inhibited in muscle cells in the high-protein level group. A high-protein diet has been linked to an increased risk of disease by activating macrophage mTOR and thus inhibiting mitochondrial autophagy, which is consistent with our findings.

## 4. Materials and Methods

### 4.1. Animals and Tissue Preparation

The healthy triploid crucian carp that were used in this experiment were purchased from the Hunan Fisheries Science Research Institute. All of the fish were placed in the experimental environment and cultured for 2 weeks. The fish were fed a commercial diet (crude protein 32.20%, crude lipid 6.54%, ash 10.4%, and gross energy 18.5 MJ/kg) twice daily at 9:00 and 15:00. Then, a certain number of healthy juvenile triploid crucian carp of similar size were selected for the experiment. All of the fish were anesthetized with 2-phenoxyethanol before being dissected. The whole dissection operation was carried out on a clean Table surface. White muscle samples were placed into a 1.5 mL EP tube and quickly inserted into liquid nitrogen for temporary storage.

### 4.2. Ingredients and Experimental Diets

Six isocaloric diets with different crude protein (CP) levels were formulated (Table 1). The diet formulation and chemical composition are shown in Table 6. The raw materials were ground separately and passed through a 40-mesh sieve (0.425 mm diameter). Subsequently, the sieved raw materials were mixed with the lipid source such as soybean oil and water with the addition order of pellet size from small to large in a mixer (model: CH-100, Jiangyin, China). After being mixed thoroughly, the mixture was extruded into 2 mm pellets by a laboratory granulator (SZLH200, Jiangsu Zhengchang Group Co., Ltd., Shanghai, China) and placed plainly on the ground out of the door to air dry. The prepared pellets were finally stored in separate sealed plastic bags at −20 °C until use.

### 4.3. Feeding Experiment Design

Six groups were set according to the kinds of dietary protein level. The experiment was carried out in the indoor breeding base of Changsha University. After 2 weeks of domestication in an indoor recirculating aquaculture system comprising 4 fiberglass tanks (1500 L), the fish (initial body weight: 11.79 ± 0.09 g) were kept in 18 fiberglass tanks (1.2 m H × 0.8 m D) (n = 30 fish in each tank). Each group comprised 3 parallel fiberglass tanks that were randomly distributed. All of the fish were adapted to the experimental diets for 1 week. During the feeding trial, the water temperature was maintained at 24.5 ± 1.0 °C, the dissolved oxygen content was kept above 6.5 mg/L, and the ammonia nitrogen concentration was <0.5 mg/L. After 8 weeks of feeding, three individuals from each fiberglass tank (n = 3 × 3) were sacrificed, and the white muscle was collected.

### 4.4. Flesh Quality Analysis

Three fish in each cage were chosen to have the dorsal muscle removed on both sides. The samples were momently pooled in a 2 mL tube frozen in liquid nitrogen, stored at −80 °C refrigerator for later analysis. The samples are placed in an oven at 105 °C and dried at a constant weight to determine the moisture content. Ash is the residue obtained after the sample is burned at high temperatures to completely escape graphite and volatiles. Crude protein content was examined by the Kjeldahl method. HPLC was employed to analyze the content of the muscle free amino acid [chromatographic column: C18 SHISEIDO (4.6 mm × 250 mm × 5 μm); detector: DAD detector; current velocity: 1.0 mL/min; column temperature: 40 °C; wavelength: 254 nm; mobile phase: A (0.1 mol/L Sodium anhydrous:acetonitrile = 97:3, pH 6.5); B (acetonitrile:water = 80:20); gradient elution (Table 1)] and flavor nucleotide [chromatographic column: Agilent C18 (4.6 mm × 250 mm × 5 μm); detector: DAD detector; current velocity:1.0 mL/min; column temperature: 25 °C; wavelength: 254 nm; mobile phase: phosphate buffer:methanol (1000:40); isocratic elution].

Two pieces of dorsal muscles per fish that were cut into a uniform thickness and size (1.0 cm × 1.0 cm × 0.25 cm) were used for the analysis of textural properties after sampling. Hardness, springiness, chewiness, adhesiveness, cohesiveness, and gumminess were determined by TPA mode (TMS-PRO, TL-Pro 1.18-408 15/10/13, FTC, Washington, DC, USA) by which the initial force was set to 0.1 N–250 N, and the probe was set up to a height of 1 cm and was lowered at a constant rate of 30 mm/min with a deformation of 60% of the original length.

### 4.5. Quantitative Real Time PCR Analysis

Total RNA was isolated using Trizol reagent (Invitrogen, Carlsbad, CA, USA). The cDNA was synthesized from 1 μg of total RNA using the Evo M-MLV RT Kit with gDNA Clean for qPCR (Accurate biology, AG11705) and were used according to the manufacturer’s instructions. The SYBR^®^ Green Premix Pro Taq HS qPCR Kit (Accurate biology, AG11701) was used for qPCR along with the Prism 7500 Sequence Detection System (Applied Biosystems, Foster City, CA, USA) to determine the mRNA expression of different genes. β-actin was used as the internal control. For each sample, three replicates were performed under the following conditions: 95 °C for 30 s followed by 40 cycles at 95 °C for 10 s, and 60 °C for 30 s. Relative mRNA expression was calculated using the 2^−ΔΔCt^ method in excel. The primers used in this study are designed with Primer Express software and shown in Table 7.

### 4.6. Western Blot Analysis

Protein expression level analysis was performed as following: Briefly, 0.1 g muscle was homogenized with 1 mL RIPA lysis buffer (Cat. No. SL1020-100 mL, Coolaber, Beijing, China) by using the homogenizer (service-bio, KZ-III-F, Wuhan, China) at 8000 rpm/min for 10 s, and repeating 3 times. The homogenized solution was stood on ice for 30 min. Then, the solution was centrifuged at 12,000 rpm/min and 4 °C for 5 min. The supernatant was transferred to a new 1.5 mL centrifuge tube. A total of 25 μL supernatant coupled with a volume of 200 μL mixture reagent was used to quantify total protein contents using BCA Protein Assay Kit (Cat. No. T9300A, Takara, Kusatsu, Japan). The proteins were quantified to the same concentration with RIPA lysis buffer and mixed with SDS-PAGE loading buffer (5×) (Cat. No. P0015L, Beyotime Institute of Biotechnology, Nantong, China) at a ratio of 5:1 and denatured for 10 min at a temperature of 95 °C or more. A total of 40 μg protein was loaded into each sampling well and separated by SDS-PAGE using the Mini-Protean Tetra Electrophoresis system (BioRad, Hercules, CA, USA), followed by transfer to polyvinylidene fluoride membranes (IPVH00010, Millipore Co., Burlington, MA, USA). After blocking non-specific binding, the membrane was incubated with the primary antibody for 120 min at room temperature before adding the HRP-conjugated secondary antibody. Finally, the BeyoECL Plus Kit (Cat. No. P0018M, Beyotime Institute of Biotechnology, Nantong, China) was used to visualize protein expression. Ponceau S was used as the loading control. Antibodies against nuclear factor erythroid 2-related factor 2 (*Nrf2*) (Cat.No.PA5-14144, Invitrogen, Waltham, MA, USA), forkhead box O1 (FoxO1) (Cat.No. PA5-23132, Invitrogen, Waltham, MA, USA) and glyceraldehyde-3-phosphate dehydrogenase (GAPDH) (Cat. No. SC-47724, Santa Cruz, CA, USA) were used.

Immunoblot signals were quantified by Image J (Version 1.8.0) [38]. Details as follows: The immunoblots was quantified by Image J and calculated by the formula [(target protein)^c^/(Ponceau S)^c^]/[(target protein)^L^/(Ponceau S)^L^]; (target protein)^c^ and (ponceau S)^c^ are the signal of target protein and ponceau S at the indicated protein level, respectively; (target protein)^L^ and (Ponceau S)^L^ are the signal of target protein and ponceau S at protein levels with lowest value of [(target protein)/(Ponceau S)], respectively.

### 4.7. Statistical Analysis

All the results are shown as the mean ± SD of different biological samples. The data were firstly subjected to normality and homoscedasticity tests and then statistical evaluation with one-way analysis of variance (ANOVA) was performed, which was followed by the Tukey multiple comparison test using SPSS 18.0. Different lowercase letters indicate significant differences (*p* < 0.05).

## 5. Conclusions

In summary, this study indicated that dietary protein levels influence muscle nutritional composition, antioxidative pathways, and autophagy levels. The 29–32% dietary protein levels would be the appropriate level range to improve muscle quality and promote the antioxidantion and autophagy capacity of triploid crucian carp muscles. So, it would provide more theoretical foundations and nutritional strategies for improving muscle quality, antioxidantion, and autophagy of triploid crucian carp.

## Figures and Tables

**Table 1:** The gradient of the mobile phase.

Time (min)	Current Velocity (mL/min)	Mobile Phase A (%)	Mobile Phase B (%)
0.0	1.0	100	0
14.0	1.0	85	15
29.0	1.0	66	34
30.0	1.0	0	100
37.0	1.0	0	100
38.0	1.0	100	0
45.0	1.0	100	0

**Table 2:** Whole body muscle composition (%) and body index of juvenile Carassius auratus triploid fed with different levels of dietary protein for 8 weeks.

	Dietary Protein Levels					
	26.00%	29.00%	32.00%	35.00%	38.00%	41.00%
Whole body						
Moisture	70.28 ± 0.44	70.48 ± 0.50	70.65 ± 0.63	70.67 ± 0.65	69.75 ± 0.65	70.24 ± 0.30
Ash	2.30 ± 0.11 ^a^	2.44 ± 0.03 ^ab^	2.39 ± 0.03 ^ab^	2.53 ± 0.04 ^b^	2.51 ± 0.06 ^b^	2.53 ± 0.05 ^b^
Crude lipid	11.25 ± 0.45	11.00 ± 0.42	10.88 ± 0.43	10.62 ± 0.42	11.26 ± 0.61	10.81 ± 0.20
Muscle						
Crude protein	19.37 ± 0.09	19.37 ± 0.27	19.47 ± 0.27	19.17 ± 0.12	19.30 ± 0.38	19.90 ± 0.35
Moisture	79.13 ± 0.73 ^ab^	79.87 ± 0.32 ^b^	78.59 ± 0.48 ^ab^	79.28 ± 0.29 ^ab^	78.51 ± 0.18 ^ab^	78.24 ± 0.21 ^a^
Ash	1.09 ± 0.03	1.02 ± 0.04	1.11 ± 0.07	1.05 ± 0.02	1.11 ± 0.05	1.15 ± 0.02
body index						
CF *	2.03 ± 0.06	2.11 ± 0.04	1.98 ± 0.08	2.10 ± 0.07	2.13 ± 0.04	2.08 ± 0.05
HIS **	10.52 ± 0.78	10.48 ± 0.53	10.44 ± 0.18	9.74 ± 0.47	10.09 ± 0.77	9.22 ± 0.49

* Condition factor (CF) = 100 × body weight (g)/body length (cm)^3^. ** Hepatopancreasomatic index (HSI) = 100 × liver weight (g)/body weight (g). Note: The different lowercase letter superscripts with in the same row represent a significant difference between the two comparison groups (*p* < 0.05). Data without superscripts indicate no significant differences (*p* > 0.05). Values are means of three replicates (n = 3 repetitions for each group with a total 3 × 3 fish).

**Table 3:** Free amino acid of muscle in juvenile Carassius auratus triploid fed with different levels of dietary protein after 8 weeks.

	Dietary Protein Levels					
	26%	29%	32%	35%	38%	41%
Flavor amino acid (mg/kg)						
Glycine	370.76 ± 26.30 ^b,^*	359.21 ± 24.85 ^b^	256.63 ± 18.03 ^a^	275.81 ± 20.50 ^a^	241.97 ± 24.11 ^a^	213.48 ± 6.16 ^a^
Alanine	251.06 ± 20.20	257.41 ± 19.16	250.35 ± 20.19	271.56 ± 14.59	271.21 ± 14.70	284.07 ± 8.98
Glutamic acid	173.12 ± 9.42 ^a^	161.18 ± 10.41 ^a^	208.89 ± 11.11 ^b^	171.99 ± 6.50 ^a^	189.48 ± 14.44 ^ab^	193.05 ± 7.99 ^ab^
Tyrosine	10.90 ± 2.86	10.46 ± 3.03	16.99 ± 4.15	10.74 ± 0.39	15.53 ± 7.25	17.70 ± 8.86
Asparagine	231.12 ± 23.03	320.48 ± 47.82	305.27 ± 16.42	253.06 ± 84.97	262.51 ± 23.35	296.27 ± 79.25
Other free amino acid (mg/kg)						
Histidine	2949.08 ± 202.38 ^a^	2922.06 ± 271.38 ^a^	3260.22 ± 75.45 ^ab^	3598.42 ± 117.00 ^b^	3639.03 ± 60.76 ^b^	3723.55 ± 100.39 ^b^
Arginine	2773.09 ± 6.90 ^ab^	2756.65 ± 75.92 ^ab^	2905.50 ± 36.70 ^bc^	2746.91 ± 36.50 ^ab^	2709.30 ± 8.88 ^a^	2953.11 ± 48.61 ^c^
Threonine	112.44 ± 7.90 ^a^	146.17 ± 10.46 ^b^	139.10 ± 8.88 ^ab^	139.12 ± 17.46 ^ab^	123.09 ± 8.72 ^ab^	122.89 ± 4.65 ^ab^
Valine	21.18 ± 7.35 ^a^	42.10 ± 4.14 ^ab^	42.10 ± 3.25 ^ab^	49.64 ± 5.32 ^b^	44.10 ± 4.68 ^b^	43.48 ± 9.03 ^ab^
Methionine	27.59 ± 2.78	29.40 ± 1.37	35.76 ± 3.05	33.33 ± 2.41	30.40 ± 2.73	30.94 ± 5.64
Isoleucine	13.74 ± 0.58 ^a^	15.88 ± 0.72 ^a^	19.36 ± 0.39 ^ab^	20.28 ± 1.54 ^ab^	18.06 ± 3.38 ^a^	25.46 ± 1.14 ^b^
Leucine	18.93 ± 6.90 ^a^	28.36 ± 1.72 ^ab^	37.56 ± 1.45 ^b^	35.69 ± 3.86 ^b^	35.97 ± 2.98 ^b^	37.45 ± 4.31 ^b^
Lysine	583.58 ± 148.72 ^a^	972.76 ± 61.65 ^b^	906.30 ± 60.35 ^b^	805.02 ± 67.35 ^ab^	906.84 ± 75.98 ^b^	965.62 ± 43.92 ^b^
Serine	51.63 ± 2.75 ^a^	99.50 ± 5.72 ^b^	74.84 ± 3.16 ^ab^	87.01 ± 0.13 ^ab^	66.47 ± 16.16 ^ab^	75.83 ± 9.36 ^ab^
Proline	164.08 ± 0.25 ^a^	280.59 ± 17.20 ^c^	276.93 ± 12.07 ^c^	234.17 ± 17.09 ^bc^	200.46 ± 30.03 ^ab^	231.17 ± 20.13 ^bc^

* The different lowercase letter superscripts with in the same row represent a significant difference between the two comparison groups (*p* < 0.05). Data without superscripts indicate no significant differences (*p* > 0.05). Values are means of three replicates (n = 3 repetitions for each group with a total 3 × 3 fish).

**Table 4:** Texture parameters of juvenile Carassius auratus triploid fed with different levels of dietary protein after 8 weeks.

	Dietary Protein Levels					
	26.00%	29.00%	32.00%	35.00%	38.00%	41.00%
Springiness	0.77 ± 0.08	0.70 ± 0.03	0.67 ± 0.04	0.74 ± 0.03	0.68 ± 0.04	0.74 ± 0.03
Hardness	18.74 ± 1.95	18.46 ± 1.39	18.65 ± 1.18	18.46 ± 1.29	19.43 ± 1.15	21.87 ± 1.55
Gumminess	3.86 ± 0.44	3.60 ± 0.37	3.10 ± 0.21	3.30 ± 0.28	3.68 ± 0.31	3.84 ± 0.32
Chewiness	3.29 ± 0.62 ^b,^*	2.62 ± 0.38 ^ab^	2.14 ± 0.25 ^a^	2.43 ± 0.23 ^ab^	2.48 ± 0.21 ^ab^	2.85 ± 0.28 ^ab^
Adhesiveness	0.036 ± 0.008	0.032 ± 0.008	0.029 ± 0.006	0.025 ± 0.006	0.026 ± 0.006	0.025 ± 0.006
Cohesiveness	0.216 ± 0.021 ^b^	0.192 ± 0.010 ^ab^	0.170 ± 0.012 ^a^	0.178 ± 0.007 ^a^	0.188 ± 0.009 ^ab^	0.174 ± 0.006 ^a^

* The different lowercase letter superscripts with in the same row represent a significant difference between the two comparison groups (*p* < 0.05). Data without superscripts indicate no significant differences (*p* > 0.05). Values are means of three replicates (*n* = 3 repetitions for each group with a total 3 × 3 fish).

**Table 5:** The Pearson’s correlation between dietary protein level and response variable.

	Pearson’s Correlation Coefficient ^a^	*p* Value (2-Tailed) ^b^
*MRF4*	0.985 **	0
Histidine	0.953 **	0.003
Glycine	−0.948 **	0.004
Adhesiveness	−0.926 **	0.008
Nrf2 protein	−0.927 **	0.008
*PRKAγ3*	−0.969 **	0.01
Alanine	0.903 *	0.014
isoleucine	0.874 *	0.023
Whole body ash	0.869 *	0.025
HSI	−0.865 *	0.026
*Nrf2*	−0.864 *	0.027
*LC3β*	0.849 *	0.032
*PRKAβ1a*	−0.840 *	0.036
FoxO1 protein	−0.822 *	0.045
Leucine	0.821 *	0.045
*GSTα*	0.820 *	0.046
AMP	0.788	0.063
*GSTθ*	−0.743	0.090
Tyrosine	0.736	0.095
*PRKAγ2b*	−0.712	0.112
Muscle moisture	−0.696	0.125
Cohesiveness	−0.683	0.135
Valine	0.68	0.138
*PRKAγ1*	−0.643	0.168
Lysine	0.587	0.220
Muscle ash	0.584	0.223
*Keap1*	0.547	0.262
*Myostatin*	0.544	0.265
GMP	0.54	0.269
Muscle protein	0.456	0.363
Glutamic	0.454	0.365
*MEF2A*	−0.441	0.381
CF	0.408	0.422
*GSTPi*	−0.385	0.451
*MyoD*	−0.373	0.467
Whole body moisture	−0.37	0.47
Whole body lipid	−0.355	0.49
Arginine	0.327	0.526
Methionine	0.318	0.539
Chewiness	−0.314	0.544
IMP	−0.314	0.544
MyHc	−0.291	0.576
CMP	0.209	0.692
Muscle *TOR*	0.203	0.699
elasticity	−0.19	0.718
*PRKAβ*	−0.182	0.73
Asparagine	0.154	0.77
*P62*	0.142	0.788
Serine	0.11	0.835
*GPX1*	−0.093	0.861
*GPX4* *α*	0.087	0.869
Threonine	−0.07	0.895
Proline	0.063	0.906
Gumminess	0.06	0.911
*LC3A*	−0.053	0.921
*PRKAα*	−0.032	0.952
*FoxO1*	0.017	0.975
*Myogenin*	0.007	0.989

^a^ Pearson’s correlation coefficient gives a value between +1 and −1 inclusive, where 1 is total positive correlation, 0 is no correlation, and −1 is total negative correlation. ^b^ *p* < 0.05 indicates significant relationship between dietary protein level and response variable (n = 6). ** indicates the extremely significant relationship (*p* < 0.01), * indicates significant relationship (*p* < 0.05), Data without “*” superscripts indicate no significant differences (*p* > 0.05) between dietary protein level and response variable.

**Table 6:** Formulation and composition of experimental diets.

	Dietary Protein Levels					
Ingredients	26%	29%	32%	35%	38%	41%
Casein	0.00	3.20	6.50	9.80	13.10	16.40
Fish meal ^a^	12.00	12.00	12.00	12.00	12.00	12.00
Soybean meal ^b^	20.00	20.00	20.00	20.00	20.00	20.00
Rapeseed meal	15.00	15.00	15.00	15.00	15.00	15.00
Fish oil	3.00	3.00	3.00	3.00	3.00	3.00
Soybean oil	3.00	3.00	3.00	3.00	3.00	3.00
Corn starch	25.00	21.00	16.80	12.60	8.40	4.20
Wheat meal	10.00	10.00	10.00	10.00	10.00	10.00
Choline	0.50	0.50	0.50	0.50	0.50	0.50
Mineral premix ^c^	3.00	3.00	3.00	3.00	3.00	3.00
Carboxymethyl Cellulose	3.00	3.00	3.00	3.00	3.00	3.00
cellulose	5.50	6.30	7.20	8.10	9.00	9.90
Total	100.00	100.00	100.00	100.00	100.00	100.00
Proximate composition						
Crude protein	26.08	29.00	32.01	35.03	38.04	41.05
Crude lipid	8.07	8.07	8.07	8.07	8.07	8.07
Moisture	9.12	6.01	10.05	8.19	5.75	7.26
Ash	6.15	6.12	6.78	6.13	6.34	6.75

^a^ Fishmeal: purchased from American Seafood Company, Seattle, WA, USA. ^b^ Rapeseed meal: purchased from Coland Feed Co., Ltd., Singapore. ^c^ Mineral premix (mg/kg diet): NaCl, 500.0; MgSO_4_·7H_2_O, 8155.6; NaH_2_PO_4_·2H_2_O, 12,500.0; KH_2_PO_4_, 16,000.0; CaH_2_PO_4_·2H_2_O, 7650.6; FeSO_4_·7H_2_O, 2286.2; C_6_H_10_CaO_6_·5H_2_O, 1750.0; ZnSO_4_·7H_2_O, 178.0; MnSO_4_·H_2_O, 61.4; CuSO_4_·5H_2_O, 15.5; CoSO_4_·7H_2_O, 0.91; KI, 1.5; Na_2_SeO_3_, 0.60; Corn starch, 899.7.

**Table 7:** Primers of real time PCR used in this study.

Primer Name	Forward Primer Sequence (5′ to 3′)	Reverse Primer Sequence (5′ to 3′)
*actin* ^a^	CTGCCCACCAACGATCTGTCCC	CTTATTTAGCCCCGCCCCCTCT
*TOR* ^b^	TCAGGGTTGTCAGCGTATTG	AGGGTTTTATGGGCTAGTGC
*IGF1* ^c^	ATTGCCCGCATCTCATCCTC	TGACCGCTAGACATCCCCTT
*MyoD* ^d^	GAAAAACCACCAACGCTGACC	CAGGATCTCCACTTTGGGCAG
*MyHc* ^e^	GTGCTTGACATTGCTGGGTT	ATGCCTTCTTTCTTGTATTCCT
*MRF4* ^f^	GTCAGTGTCTAATGTGGGCTTG	GGATTGGGCACCGTCTTTTTCT
*Myogenin*	TCTTCGCAGACCAGCGTTTTT	CAACCCCACTCCGTTTGACAG
*Myostatin*	GTTCTGGGGGATGACAGTAAGG	TTGAACGATGGGGTCAGGCTCT
*MEF2A* ^g^	AAGAAGATACAGATCACACGG	TGCTGGCATACTGAAAGAG
^h^ *GSTα*	ACTCTCAGTTCCTGGTGGGA	CGCCTGGATTTTGGGAAAGG
*GSTθ*	TGCAAGACAGACCGTTCGTT	TCCATGCCTTGAGTTTGGGT
*GSTPi*	TGCCCAAACACCTCAAACCA	CAGCAGAAGGTCGAACAGGT
^i^ *GPX1*	CGGACATCAGGAGAACACCA	ACACCGTTCACTTCCAGCTT
*GPX4α*	CTGTGGAAGTGGCTGAAGGA	GCGGGGAGTATCTCATCACA
*Nrf2* ^j^	CGGTTTTAGCGACTCCGACT	TTCATCTCCTGCTGCTCTGC
*Keap1* ^k^	ACGCCCAGAGCAAAGACTAC	TAGCTCAGAGACTGCCGGAA
^l^ *PRKAα2*	TACCGTGCCATGAAACAGCT	GCTACGATTGTCCACCTGGT
*PRKAβ1α*	GTGATCCAGCTTTGCTCCCT	TGTACAGCAGGGTTGTGACA
*PRKAβ2*	TTGTCACCAGTCAGATGGGC	AGGTGGAGAGCTGGACAGAT
*PRKAγ1*	TAAAGCACTCCAGCATCGCT	AACCTGTGAACCTCAGCCTC
*PRKAγ2b*	CAAACCCCCTATCGCCTTGT	TCACTGTCTGGCTCTTCAGC
*PRKAγ3*	ATCAGCGAGCAAGGGAAAGT	GTAACACTTCAGCACCCCCT
^m^ *LC3A*	AGCAACTTCCCGTCTTGGAC	ACTGACCATGCTGTGCTGAT
*LC3β*	TTCCTGCTTGTCAACGGTCA	TGTCTCCTGGGATGCGTAGA
*FoxO1* ^n^	CTCCCACAGCAACGATGACT	TCAGAGTCGCCAAGTTCGTC
*p62* ^o^	AACATGAGAGAGGACGGTGC	ACTTTGCTACGCTTCCCCTC

Note: ^a^ β-actin: beta-actin; ^b^ TOR: target of rapamycin; ^c^ IGF1: Insulin-like growth factor 1; ^d^ MyoD: myogenic differentiation; ^e^ *MyHc*: Beta-Myosin Heavy Chain; ^f^ *MRF4*: myogenic regulatory factor 4; ^g^ MEF2A: myocyte enhancer factors 2; ^h^ GST: glutathione S-transferase; ^i^ *GPX:* glutathione Peroxidase; ^j^ *Nrf2*: NF-E2-related nuclear factor 2; ^k^ *Keap1*: kelch-like ECH-associated protein 1; *^l^ PRKA*:protein kinase, AMP-activated; ^m^ *LC3*: microtubule-associated protein 1 light chain 3; ^n^ *FoxO1*: forkhead box O 1; ^o^ *p62*: sequestosome 1.

## Data Availability

The datasets used and/or analyzed during the current study are available from the corresponding author on reasonable request.
